# Cabazitaxel in recurrent/metastatic squamous cell carcinoma of the head and neck: phase II UNICANCER trial ORL03

**DOI:** 10.18632/oncotarget.15901

**Published:** 2017-03-04

**Authors:** Jerome Fayette, Joel Guigay, Christophe Le Tourneau, Marian Degardin, Frederic Peyrade, Eve-Marie Neidhardt, Marie-Paule Sablin, Caroline Even, Florence Orlandini, Béata Juzyna, Carine Bellera

**Affiliations:** ^1^ Léon Bérard Center, University of Lyon, Lyon, France; ^2^ Gustave Roussy Institute, Villejuif, France; ^3^ Antoine Lacassagne Center, Nice, France; ^4^ Institut Curie, Saint-Cloud & Paris, France; ^5^ EA7285, Versailles-Saint-Quentin-en-Yvelines University, Montigny-le-Bretonneux, France; ^6^ Oscar Lambret Center, Lille, France; ^7^ Paul Strauss Center, Starsbourg, France; ^8^ H&N Unicancer Group, Paris, France; ^9^ Bergonié Institute, Clinical and Epidemiological Research Unit & INSERM U897 & Data Center for Cancer Clinical Trials, Bordeaux, France

**Keywords:** Cabazitaxel, head and neck cancer, recurrent, platinum failure

## Abstract

Treatments are limited after platinum Cetuximab or anti-PD1 failure for patients with recurrent/metastatic head and neck squamous cell carcinoma. Cabazitaxel has increased overall survival in hormone-refractory metastatic prostate cancer after failure of Docetaxel. Our aim was to detect a signal of activity with Cabazitaxel in patients with head and neck cancer who had failed platinum-, Cetuximab- and taxanes-based chemotherapy.

This multicenter phase II trial included progressive patients with an ECOG ≤2. Cabazitaxel was given at 25 mg/m^2^/3 weeks (maximum of 10 cycles), with growth factors support. Efficacy was centralized and assessed every 6 weeks. The primary endpoint was control rate at six-weeks. A Simon’s two-stage optimal design (P0=0.10; P1=0.30) required 29 evaluable patients. At the end of trial, at least 6 non-progressions were required to consider the drug worthy of further study.

Out of the 31 enrolled patients, 29 were eligible; 42% had received at least three previous lines of chemotherapy. For the primary end point, 8 patients (27.6%; 95%CI 12.7%-47.2%) had a stable disease at six weeks. Median progression-free survival was 1.05 months (95%CI 0.69-2.07). All patients were analyzed for toxicity: 6 patients had febrile neutropenia.

During the 81 cycles administered, 49 grade 3-5 events were observed concerning 81% of the patients, including 35 severe adverse events of which 15 were related to Cabazitaxel.

Although Cabazitaxel met its primary endpoint to deserve further investigations, its toxicity makes it difficult to use in frail patients and new schemes are needed (20 mg/m^2^ for example) if further investigations are launched.

## INTRODUCTION

Patients with either recurrent or metastatic (R/M) head and neck squamous cell carcinoma (HNSCC) have a poor prognosis. Standard of care combines Cisplatin, Fluorouracil and Cetuximab (optional). The median overall survival varies between from 9 and to 11 months [1, 2]. Before immunotherapy, the only approved drugs in second line were Methotrexate or Cetuximab with a median overall survival of about 6 months [3, 4]. Identifying potential new drugs is needed.

Taxanes as induction chemotherapy increased overall survival in locally advanced HNSCC [5–7]. They showed efficacy for R/M HNSCC [8–13]

Cabazitaxel is a tubulin-binding taxane drug as potent as Docetaxel in cell lines and with antitumour activity in models resistant to Paclitaxel and Docetaxel [14]. Neutropenia in phase I and II was the primary toxicity, and the recommended doses were 20 and 25 mg/m^2^ every 3 weeks. In castration-resistant prostate cancer, Cabazitaxel is active in patients who have progressed on Docetaxel [15].

We hypothesized that Cabazitaxel might be effective in HNSCC after failure of taxanes. Our objective in this phase II trial was to detect a signal of activity in a small study and to evaluate the safety of Cabazitaxel in R/M HNSCC after the failure of platinum, Cetuximab and taxanes.

## MATERIALS AND METHODS

### Study design and patient selection

ORL03 is a multicenter single-arm phase II trial. Eligible patients were aged ≥ 18 years with R/M squamous cell carcinomas of the oral cavity, oropharynx, hypopharynx or larynx, not amenable for salvage surgery or radiotherapy. Eligible patients had an ECOG performance status (PS) of 0 - 2, a documented evidence of progression based on the investigator's assessment (measurable disease according to Response Evaluation Criteria in Solid Tumors version 1·1 [RECIST v1·1]) following platinum, anti-EGFR therapy, and taxanes (19 patients received Docetaxel and 12 Paclitaxel) for R/M disease. They had not progressed within 3 months after the end of treatment with curative intent.

The study protocol was designed in accordance with the Declaration of Helsinki and the International Conference on Harmonization Guideline for Good Clinical Practice, and was approved by Independent Ethics Committees for each center. All patients provided a written informed consent for trial participation.

### Procedures

Cabazitaxel was provided by Sanofi (Paris, France). It was administered intravenously at an initial dose of 25 mg/m^2^ over 1 hour every 3 weeks up to 10 cycles. Primary prophylaxis with granulocyte-colony stimulating factor (G-CSF) was mandatory. In the case of toxicity the dose could be reduced to 20 mg/m^2^. No further dose reduction was allowed and patients were withdrawn after recurrent toxicity.

Tumor assessments were performed every 6 weeks by computed tomography imaging.

### Outcomes

The primary endpoint was the control rate (according to RECIST 1.1) at six weeks, defined as patients with objective response (complete or partial) or stable disease. All successes were reviewed by independent experts.

Secondary endpoints were overall response rate (ORR) at six weeks, progression-free survival (PFS), overall survival (OS), as well as safety and health-related quality of life (HRQoL). PFS was defined as the delay between inclusion and progression or death. OS was defined as the delay between inclusion and death. Safety was assessed for each cycle using the National Cancer Institute Common Terminology Criteria for Adverse Events Version 3.0 (NCI CTC-AE v3).

Patient-reported outcomes were assessed using the European Organization for Research and Treatment of Cancer (EORTC) Quality of Life Questionnaire (QLQ-C30) and the head and neck cancer-specific supplementary module (QLQ-H&N35) at the onset of treatment, at third cycle and at the end of treatment.

### Statistical analysis

A Simon's optimal two-stage design was used with the following assumptions: 10% undesirable six-week control rate (null hypothesis, corresponding to what is reported with taxanes in second-line for R/M HNSCC), 30% target six-week control rate, 5% type 1 error rate and 80% power. A total of 29 eligible and evaluable subjects was re required for efficacy analysis (31 were included to take into account ineligible patients). Based on the first stage of Simon's design, 10 patients had to be enrolled with at least 2 non-progressions to be able to proceed for the second stage. At the end of the second stage (*N* = 29), at least 6 non-progressions were required in order to make the drug worthy of further study (six-week control rate considered to be > 10%). To be evaluable for efficacy, a subject had to meet the eligibility criteria and receive at least one treatment administration. Safety data were reported in the safety population defined as any patient with at least one treatment injection.

Descriptive statistics were used to characterize patients at the start of the study. 95% two-sided confidence intervals (CI, binomial law) were computed for control rate and objective response rates. PFS and OS were estimated using the Kaplan-Meier method. HRQoL data were analysed following the EORTC recommendations. Data reported here represent the study database on 23 October 2014. All analyses were carried out with SAS 9·2 (SAS Institute, Cary, NC).

## RESULTS

### Patients and treatment exposure

Between April 2012 and April 2013, 31 patients were included in 5 centers in France. Inclusions were suspended between May 2012 and October 2012 for interim analysis. Two patients were considered as non-eligible (progression within 3 months after initial curative treatment, use of Carbamazepine, which was not allowed because of its interaction with Cytochrome P450). Patients characteristics are summarized in Table 1. They were heavily pretreated.

**Table 1:** Patient demographics and baseline characteristics

Characteristics of all included patients	*N* (%)
**Sex** Male Female	24 (77%) 7 (23%)
**Initial location of the tumor** Oral cavity Oropharynx Hypopharynx Larynx	10 (32%) 14 (45%) 4 (13%) 3 (10%)
Stage at the initial diagnosis I II III Iva IVb IVc Unknown	2 (6%) 3 (10%) 6 (19%) 14 (45%) 3 (10%) 2 (6%) 1 (3%)
**Initial curative treatment (all but IVc patients)** Surgery then Radiotherapy Chemoradiotherapy Surgery then Chemoradiotherapy	7 (24%) 14 (48%) 8 (28%)
**Chemotherapies for recurrent/metastatic disease** **First line** Platin-Taxane-Cetuximab Platin-Taxane Platin-5FU Platin-Taxane-5FU Platin-Cetuximab Platin-5FU-Cetuximab Taxane **Second line** Cetuximab (or other anti-EGFR) Taxane-Cetuximab Methotrexate Platin-Taxane Platin-5FU-Cetuximab Platin-Taxane-Cetuximab Taxane Capecitabine Etoposide **Third line** Capecitabine Platin-Taxane Cetuximab Platin-Cetuximab Taxane Gemcitabine Clinical Trial **Fourth line** Methotrexate Platin-Cetuximab Taxane Capecitabine Vinorelbine **Fifth line** Methotrexate Taxane	31 patients 10 (32%) 7 (23%) 4 (13%) 4 (13%) 3 (10%) 2 (6%) 1 (3%) 29 patients (94%) 10 (35%) 6 (21%) 5 (17%) 3 (10%) 1 (3%) 1 (3%) 1 (3%) 1 (3%) 1 (3%) 13 patients (42%) 5 (38%) 2 (15%) 2 (15%) 1 (8%) 1 (8%) 1 (8%) 1 (8%) 8 patients (26%) 3 (38%) 2 (25%) 1 (12%) 1 (12%) 1 (12%) 2 patients (6%) 1 (50%) 1 (50%)
**Disease at the onset of Cabazitaxel** Locoregional recurrence only Locoregional recurrence with metastases Metastases only	9 (29%) 13 (42%) 9 (29%)
**Age at the onset of Cabazitaxel**	Median 60 years [30–71]
**Performance status at the onset of Cabazitaxel** 0 1 2	4 (13%) 19 (61%) 8 (26%)

The median follow-up was 442 days, with only one patient lost to follow-up. The median number of cycles was 2 [range 1-6] and a total of 81 treatment cycles were administered.

### Efficacy

In the interim analysis, 9/10 patients were eligible for efficacy assessment and 2 underwent independently reviewed stabilization at six weeks. The study continued into the second step. The final analysis of the 29 eligible patients showed 8 stabilizations at 6 weeks, and 21 progressions. At 12 weeks, 5 patients were stable and all progressed at 18 weeks. No tumor shrinkage was observed. With 8 non-progressive patients at 6 weeks, six-week control rate was 27.6% (95%CI 12.7%-47.2%). Therefore the primary endpoint was met with a six-week control rate > 10% as initially targeted.

**Figure 1:**
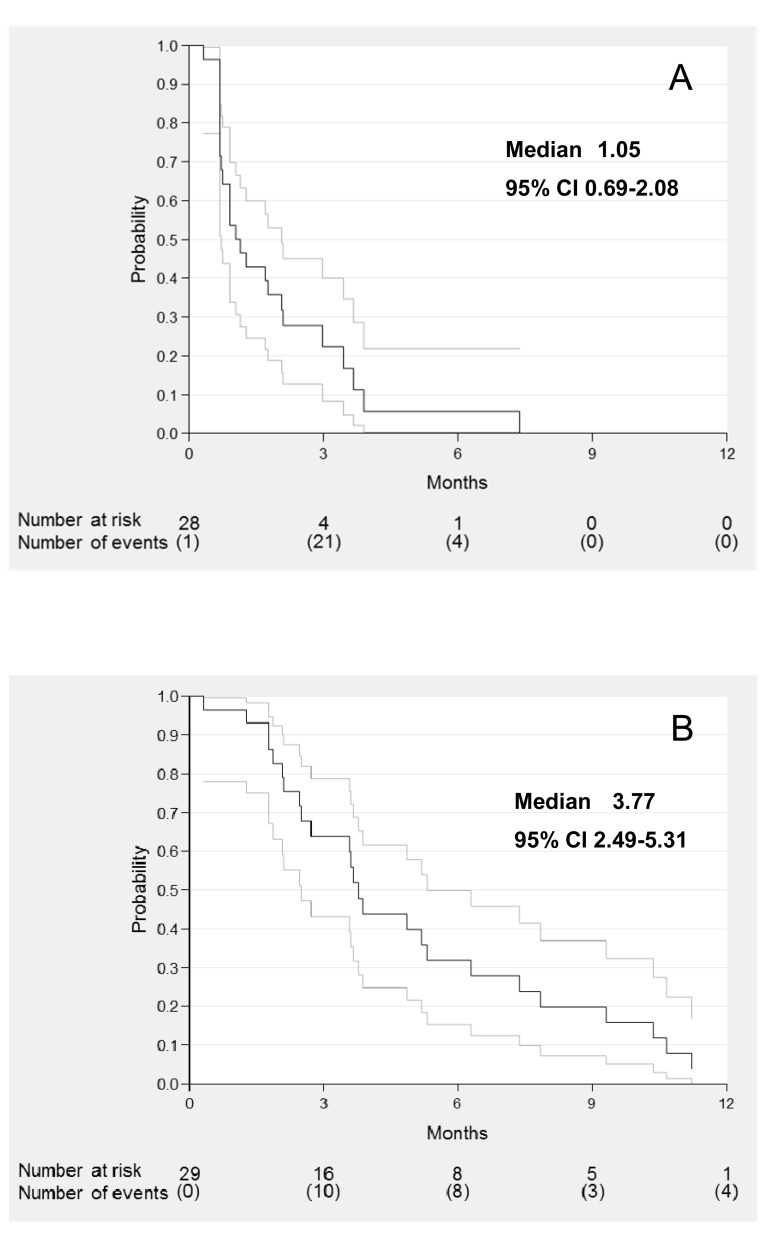
Progression free survival A. and overall survival B. of evaluable patients

Median PFS was 1.05 months (95%CI 0.69-2.07) (Figure 1A) and median OS was 3.77 months (95%CI 2.49-5.31) (Figure 1B).

### Safety

Toxicities are detailed in Table 2. The main toxicities were hematological: 6 (19%) febrile neutropenia (one fatal after the sixth cycle) despite primary prophylaxis with G-CSF. Concerning grade 3-4 non-hematological toxicities (13 patients, 42%), we observed pulmonary toxicities, frequently observed with head and neck cancers, with 3 dyspnea (10%) and 7 sepsis (23%), mostly pneumonitis.

**Table 2:** Toxicities observed during the treatment by Cabazitaxel (*N* = 31 patients)

Toxicities, *N* patients (%)	Grade 1-2	Grade 3-5
Febrile Neutropenia	-	**6** (19%; 1 death at cycle 6)
Neutropenia	**2** (6%)	**5** (16%)
Lymphopenia	**7** (23%)	**12** (39%)
Thrombopenia	**3** (10%)	**5** (16%)
Anemia	**17** (55%)	**6** (19%)
Diarrhea	**7** (23%)	**2** (6%)
Nausea	**10** (32%)	-
Mucositis	**6** (19%)	-
Neuropathy	**4** (13%)	-
Pneumopathy/ Sepsis	**1** (3%)	**7** (23%)
Dyspnea	**3** (10%)	**3** (10%)
Asthenia	12 (39%)	2 (6%)

After 81 treatment cycles administered, 35 Serious Adverse Events were reported (15 related to Cabazitaxel).

Three patients (10%) needed a dose-reduction. In addition to the patient who died from febrile neutropenia, one patient stopped treatment because of toxicity. All other patients stopped treatment because of disease progression.

### Patient-reported outcomes

**Figure 2:**
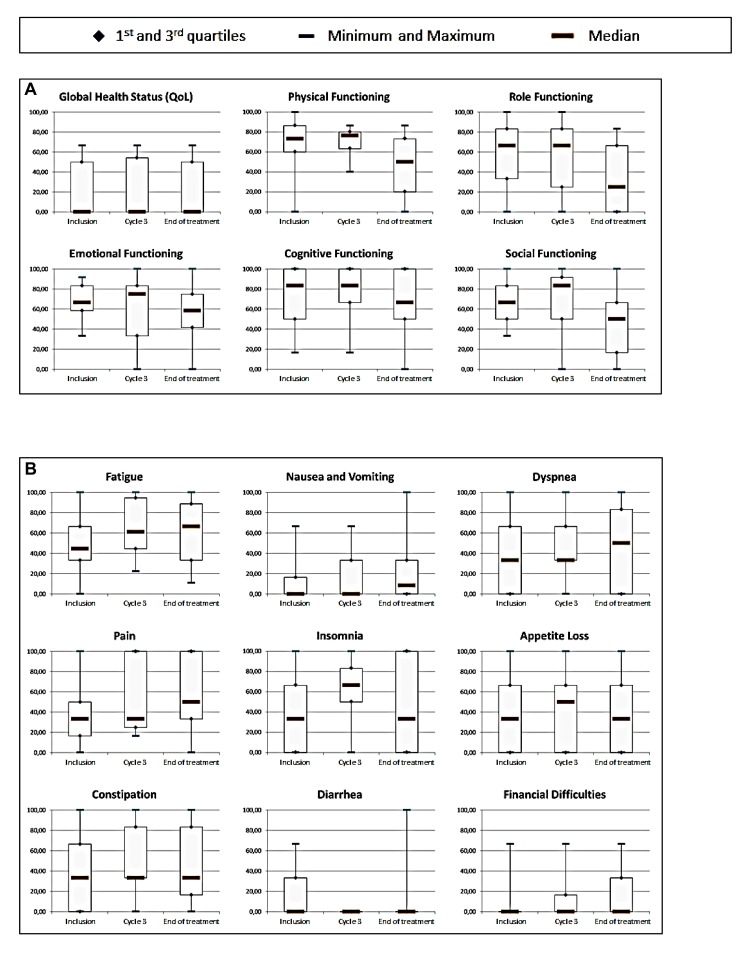

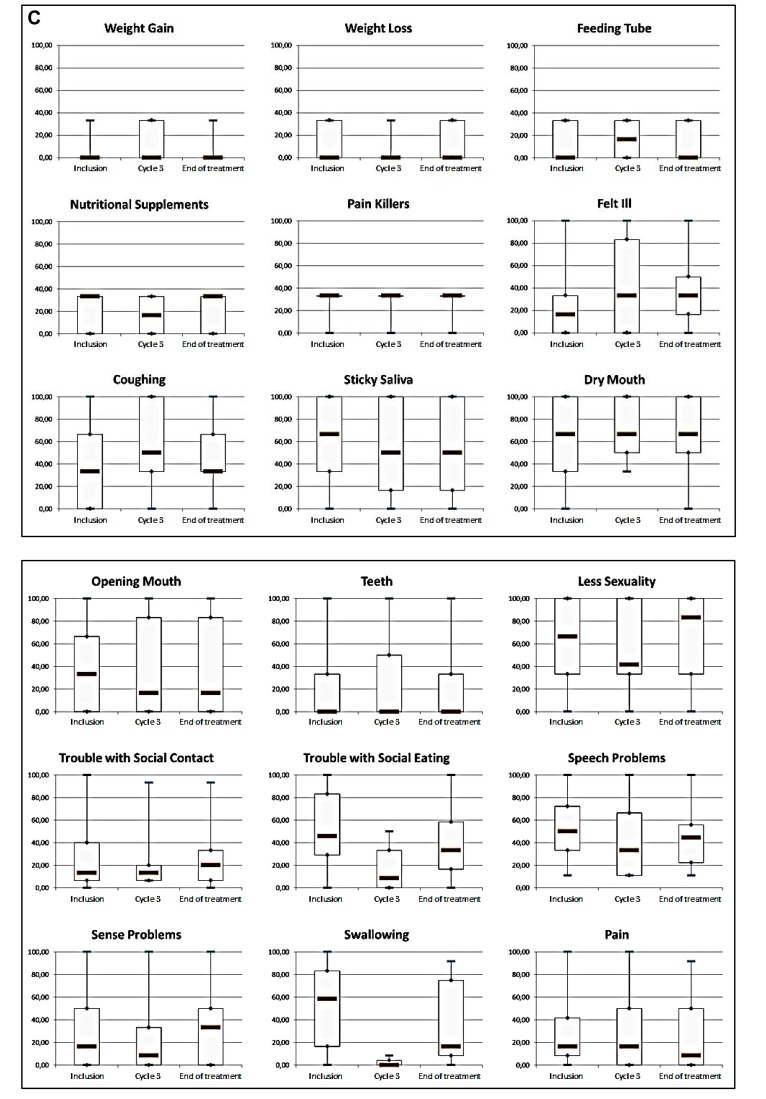
Health-related quality of life at baseline, 3 cycles and end of treatment based on the EORTC QLQ-C30 functional domains A., QLQ-C30 symptomatic scales B. and complementary module H&N35 C

All patients completed the EORTC questionnaires at baseline, 8/9 patients were still on treatment at the third cycle and 16/31 at the end of treatment completed the questionnaires. As shown in Figure 2, no significant change in quality of life with Cabazitaxel was observed. The global health status (QoL) at baseline was poor. A deterioration of the functional scales of the QLQ-C30 was observed at the end of the treatment. A non-significant improvement of the scores for fatigue, insomnia and appetite loss in the symptomatic scales of the QLQ-C30 were observed. Similarly, no clear changes were noted in the items of the complementary questionnaire H&N35, except for a degradation of the swallowing capacities during the third cycle.

## DISCUSSION

Cabazitaxel met its primary endpoint with 8/29 (27.6% [95%CI 12.7%-47.2%]) non-progressive patients at six weeks, more than six patients required. However, PFS and OS seem short and the tolerance poor. But our patients were heavily pretreated with advanced and symptomatic resistant disease. The objective of this trial was to detect a signal of activity of Cabazitaxel in order to determine whether it was of interest in head and neck cancers. We would like to discuss two points:

Is the control rate at six-weeks appropriate to detect a potentially effective drug in HNSCC?

Are the results sufficient to justify future trials in patients with HNSCC?

1. This type of tumor usually progresses rapidly with short survival. The “Extreme” schedule, (Cisplatin, 5-fluorouracile and Cetuximab) allows a PFS of 5.6 months and an OS of 10.1 months [1]. With Methotrexate, the standard second-line, PFS is 1.7 months [4]. Thus for our heavily pretreated patients (before Cabazitaxel, 42% received ≥ 3 lines for recurrent disease, and 26% ≥ 4 lines), a six-week control rate is an appropriate endpoint. All patients were progressive before inclusion. The validity of this endpoint was confirmed by comparison with Capecitabine (an oral prodrug of fluorouracile) in this situation. Indeed, since fluorouracile was effective as a single agent [16] and that, after failure of platinum, Capecitabine showed 24.2% response [17], we retrospectively demonstrated in 29 heavily pretreated patients (86% ≥ 3 lines) 2 months of PFS and 14 responses or stabilizations with Capecitabine [18]. We therefore conclude that an effective drug in HNSCC can be detected by our design.
2. The efficacy results should not be over-interpreted. The short PFS and OS were expected in this population resistant to chemotherapy and of poor prognosis. No other effective drug was available. As indicated, Methotrexate in second-line gives a PFS of 1.7 months and an OS of 6.2 months [4]. With 1.05 months of PFS and 3.77 months of OS for resistant patients (they received platinum, Cetuximab, and taxanes and 8/29 (28%) had also Methotrexate), we can say that Cabazitaxel is of interest. This trial cannot be compared to other studies investigating the efficacy in second-line: our objective was only to detect a potential drug deserving further investigations.

To our knowledge, two other studies investigated Cabazitaxel in HNSCC. First, a randomized phase II trial with 101 patients in second-line compared Cabazitaxel to Methotrexate [19]. The PFS at 18 weeks (primary endpoint) was not significantly different with Cabazitaxel (13.2%) and Methotrexate (8.5%). A similar PFS was observed at 1.9 months, but with a trend in OS in favor of Cabazitaxel with 5 months *versus* 3.6 months and at least Cabazitaxel did not seem inferior to an approved drug such as Methotrexate [4]). The second phase I study of 13 patients in induction with Cisplatin, Fluorouracile and Cabazitaxel (instead of Docetaxel) showed 90% response without any particular toxicity, suggesting the efficacy of Cabazitaxel in chemo-naive patients [20]. The data from these studies and from our own study could support the potential interest of Cabazitaxel in the treatment of HNSCC.

One patient died of febrile neutropenia at the sixth cycle, another stopped the treatment due to toxicity, and three needed dose reductions. The major hematological toxicities were expected. In the pivotal study in prostate cancer, the most common clinically significant grade 3 or higher toxicities were neutropenia (82%), febrile neutropenia (8%), and diarrhea (6%) [15]. The majority of adverse events were related to the cancer itself: lung infections related to swallowing disorders (common and serious in HNSCC), anemia and lymphopenia.

To sum up, Cabazitaxel gave a signal of activity in HNSCC but was toxic. In future studies, Cabazitaxel could be used at 20 mg/m^2^ every 3 weeks or weekly at a lower dose like Paclitaxel or Docetaxel. Comparisons with Docetaxel in induction or in third line after platin-based chemotherapy and immunotherapy could be performed. Clearly Cabazitaxel with its toxicity and results could not replace immunotherapy after failure of platinum agent since Nivolumab demonstrated its superiority in terms of efficacy and tolerability compared to chemotherapy in this situation [21]. Furthermore, this study validates an efficient strategy in terms of clinical trial design for the detection of potential drugs in HNSCC.

## References

[1] Vermorken JB, Mesia R, Rivera F, Remenar E, Kawecki A, Rottey S, Erfan J, Zabolotnyy D, Kienzer HR, Cupissol D, Peyrade F, Benasso M, Vynnychenko I (2008). Platinum-based chemotherapy plus cetuximab in head and neck cancer. N Engl J Med.

[2] Vermorken JB, Stöhlmacher-Williams J, Davidenko I, Licitra L, Winquist E, Villanueva C, Foa P, Rottey S, Skladowski K, Tahara M, Pai VR, Faivre S, Blajman CR (2013). Cisplatin and fluorouracil with or without panitumumab in patients with recurrent or metastatic squamous-cell carcinoma of the head and neck (SPECTRUM): an open-label phase 3 randomised trial. Lancet Oncol.

[3] Vermorken JB, Trigo J, Hitt R, Koralewski P, Diaz-Rubio E, Rolland F, Knecht R, Amellal N, Schueler A, Baselga J (2007). Open-label, uncontrolled, multicenter phase II study to evaluate the efficacy and toxicity of cetuximab as a single agent in patients with recurrent and/or metastatic squamous cell carcinoma of the head and neck who failed to respond to platinum-based therapy. J Clin Oncol.

[4] Machiels JP, Haddad RI, Fayette J, Licitra LF, Tahara M, Vermorken JB, Clement PM, Gauler T, Cupissol D, Grau JJ, Guigay J, Caponigro F, de Castro G (2015). Afatinib versus methotrexate as second-line treatment in patients with recurrent or metastatic squamous-cell carcinoma of the head and neck progressing on or after platinum-based therapy (LUX-Head & Neck 1): an open-label, randomised phase 3 trial. Lancet Oncol.

[5] Hitt R, López-Pousa A, Martínez-Trufero J, Escrig V, Carles J, Rizo A, Isla D, Vega ME, Martí JL, Lobo F, Pastor P, Valentí V, Belón J (2005). Phase III study comparing cisplatin plus fluorouracil to paclitaxel, cisplatin, and fluorouracil induction chemotherapy followed by chemoradiotherapy in locally advanced head and neck cancer. J Clin Oncol.

[6] Posner MR, Hershock DM, Blajman CR, Mickiewicz E, Winquist E, Gorbounova V, Tjulandin S, Shin DM, Cullen K, Ervin TJ, Murphy BA, Raez LE, Cohen RB (2007). Cisplatin and fluorouracil alone or with docetaxel in head and neck cancer. N Engl J Med.

[7] Vermorken JB, Remenar E, van Herpen C, Gorlia T, Mesia R, Degardin M, Stewart JS, Jelic S, Betka J, Preiss JH, van den Weyngaert D, Awada A, Cupissol D (2007). Cisplatin, fluorouracil, and docetaxel in unresectable head and neck cancer. N Engl J Med.

[8] Argiris A, Ghebremichael M, Gilbert J, Lee JW, Sachidanandam K, Kolesar JM, Burtness B, Forastiere AA (2013). Phase III randomized, placebo-controlled trial of docetaxel with or without gefitinib in recurrent or metastatic head and neck cancer: an eastern cooperative oncology group trial. J Clin Oncol.

[9] Gibson MK, Li Y, Murphy B, Hussain MH, DeConti RC, Ensley J, Forastiere AA, Eastern Cooperative Oncology Group (2005). Randomized phase III evaluation of cisplatin plus fluorouracil versus cisplatin plus paclitaxel in advanced head and neck cancer (E1395): an intergroup trial of the Eastern Cooperative Oncology Group. J Clin Oncol.

[10] Grau JJ, Caballero M, Verger E, Monzó M, Blanch JL (2009). Weekly paclitaxel for platin-resistant stage IV head and neck cancer patients. Acta Otolaryngol.

[11] Guardiola E, Peyrade F, Chaigneau L, Cupissol D, Tchiknavorian X, Bompas E, Madroszyk A, Ronchin P, Schneider M, Bleuze JP, Blay JY, Pivot X (2004). Results of a randomised phase II study comparing docetaxel with methotrexate in patients with recurrent head and neck cancer. Eur J Cancer.

[12] Guigay J, Fayette J, Dillies AF, Sire C, Kerger JN, Tennevet I, Machiels JP, Zanetta S, Pointreau Y, Bozec Le Moal L, Henry S, Schilf A, Bourhis J (2015). Cetuximab, docetaxel, and cisplatin as first-line treatment in patients with recurrent or metastatic head and neck squamous cell carcinoma: a multicenter, phase II GORTEC study. Ann Oncol.

[13] Hitt R, Amador ML, Quintela-Fandino M, Jimeno A, del Val O, Hernando S, Cortes-Funes H (2006). Weekly docetaxel in patients with recurrent and/or metastatic squamous cell carcinoma of the head and neck. Cancer.

[14] Vrignaud P, Sémiond D, Lejeune P, Bouchard H, Calvet L, Combeau C, Riou JF, Commerçon A, Lavelle F, Bissery MC (2013). Preclinical antitumor activity of cabazitaxel, a semisynthetic taxane active in taxane-resistant tumors. Clin Cancer Res.

[15] de Bono JS, Oudard S, Ozguroglu M, Hansen S, Machiels JP, Kocak I, Gravis G, Bodrogi I, Mackenzie MJ, Shen L, Roessner M, Gupta S, Sartor AO, TROPIC Investigators (2010). Prednisone plus cabazitaxel or mitoxantrone for metastatic castration-resistant prostate cancer progressing after docetaxel treatment: a randomised open-label trial. Lancet.

[16] Jacobs C, Lyman G, Velez-García E, Sridhar KS, Knight W, Hochster H, Goodnough LT, Mortimer JE, Einhorn LH, Schacter L (1992). A phase III randomized study comparing cisplatin and fluorouracil as single agents and in combination for advanced squamous cell carcinoma of the head and neck. J Clin Oncol.

[17] Martinez-Trufero J, Isla D, Adansa JC, Irigoyen A, Hitt R, Gil-Arnaiz I, Lambea J, Lecumberri MJ, Cruz JJ (2010). Phase II study of capecitabine as palliative treatment for patients with recurrent and metastatic squamous head and neck cancer after previous platinum-based treatment. Br J Cancer.

[18] Péron J, Poupart M, Ceruse P, Ramade A, Girodet D, Zrounba P, Fayette J (2012). Efficacy and safety of capecitabine in heavily pretreated recurrent/metastatic head and neck squamous cell carcinoma. Anticancer Drugs.

[19] Rottey S, Van Maanen A, Vandenbulcke JM, Filleul B, Seront E, Henry S, D’Hondt LA, Lonchay C, Hollbrechts S, Boegner P, Brohee DJ, Dequanter D, Louviaux I (2015). Randomized phase II study of cabazitaxel versus methotrexate in patients with recurrent or metastatic squamous cell carcinoma of the head and neck (SCCHN) previously treated with platinum-based therapy. J Clin Oncol (ASCO meeting).

[20] Khan A, Babu R, Vaz A, Deyne-Borza M, Price A, Lorne Bakst R, Gupta V, Miles B, Posner MR, Misiukiewicz K (2015). Phase I study of cabazitaxel-PF induction chemotherapy in patients with locally advanced squamous cell carcinoma of the head and neck (SCCHN). J Clin Oncol (ASCO meeting).

[21] Ferris RL, Blumenschein G, Fayette J, Guigay J, Colevas AD, Licitra L, Harrington K, Kasper S, Vokes EE, Even C, Worden F, Saba NF, LC Iglesias Docampo (2016). Nivolumab for Recurrent Squamous-Cell Carcinoma of the Head and Neck. N Engl J Med.

